# Quantifying the effect of Vpu on the promotion of HIV-1 replication in the humanized mouse model

**DOI:** 10.1186/s12977-016-0252-2

**Published:** 2016-04-18

**Authors:** Hiroki Ikeda, Shinji Nakaoka, Rob J. de Boer, Satoru Morita, Naoko Misawa, Yoshio Koyanagi, Kazuyuki Aihara, Kei Sato, Shingo Iwami

**Affiliations:** Department of Biology, Faculty of Sciences, Kyushu University, 6-10-1 Hakozaki, Higashi-ku, Fukuoka, Fukuoka 812-8581 Japan; Graduate School of Medicine, University of Tokyo, Tokyo, Japan; Theoretical Biology, Utrecht University, Utrecht, The Netherlands; Department of Mathematical and Systems Engineering, Shizuoka University, Shizuoka, Japan; Laboratory of Viral Pathogenesis, Institute for Virus Research, Kyoto University, 53 Shogoinkawara-cho, Sakyo-ku, Kyoto, Kyoto 606-8507 Japan; Institute of Industrial Science, The University of Tokyo, Tokyo, Japan; Graduate School of Information Science and Technology, The University of Tokyo, Tokyo, Japan; CREST, JST, Saitama, Japan; PRESTO, JST, Saitama, Japan

**Keywords:** Virus dynamics, Mathematical model, Vpu, Tetherin, HIV-1, Humanized mouse model

## Abstract

**Background:**

Tetherin is an intrinsic anti-viral factor impairing the release of nascent HIV-1 particles from infected cells. Vpu, an HIV-1 accessory protein, antagonizes the anti-viral action of tetherin. Although previous studies using in vitro cell culture systems have revealed the molecular mechanisms of the anti-viral action of tetherin and the antagonizing action of Vpu against tetherin, it still remains unclear how Vpu affects the kinetics of HIV-1 replication in vivo.

**Results:**

To quantitatively assess the role of Vpu in viral replication in vivo, we analyzed time courses of experimental data with viral load and target cell levels in the peripheral blood of humanized mice infected with wild-type and *vpu*-deficient HIV-1. Our recently developed mathematical model describes the acute phase of this infection reasonably, and allowed us to estimate several parameters characterizing HIV-1 infection in mice. Using a technique of Bayesian parameter estimation, we estimate distributions of the basic reproduction number of wild-type and *vpu*-deficient HIV-1. This reveals that Vpu markedly increases the rate of viral replication in vivo.

**Conclusions:**

Combining experiments with mathematical modeling, we provide an estimate for the contribution of Vpu to viral replication in humanized mice.

**Electronic supplementary material:**

The online version of this article (doi:10.1186/s12977-016-0252-2) contains supplementary material, which is available to authorized users.

## Findings

Tetherin is also known as bone marrow stromal antigen 2 (BST2), CD317, and HM1.24, and impairs the release of a broad range of enveloped viruses including human immunodeficiency virus type 1 (HIV-1) by tethering budded virions on the surface of infected cells [1–3]. HIV-1 expresses the accessory protein Vpu which antagonizes the anti-viral effect of tetherin [1–3]. Previous studies using in vitro cell culture systems have revealed the molecular mechanisms of tetherin-mediated anti-viral effect and its antagonism by Vpu [3–5]. We have recently performed experiments using human hematopoietic stem cell-transplanted humanized mouse model infected with wild-type and *vpu*-deficient HIV-1 (HIV-1Δ*vpu* [6, 7]). These findings suggested that Vpu counteracts the anti-viral effect of tetherin in infected humanized mice, augmenting HIV-1 replication in vivo [8]. We investigated and quantified the role of Vpu during the acute phase of infection using the humanized mouse model, which reasonably mimics HIV-1 infection in human before the onset of adaptive immune responses [8, 9].

To quantitatively understand the effect of Vpu on dynamics of viral replication and infection in vivo, we here perform mathematical modeling of the experimental data published in [8]. Previously we simplified a “basic model” of virus infection [10, 11], to derive a novel model that can be fitted to time course data from HIV-1 infected humanized mice and reliably estimate the parameters characterizing the dynamics of an acute infection (see Additional file 1) [12, 13]:

1 $$ \frac{dT\left( t \right)}{dt} = - \beta T\left( t \right)V\left( t \right), $$

2 $$ \frac{dV\left( t \right)}{dt} = rT\left( t \right)V\left( t \right) - \delta V\left( t \right). $$

Here $T\left( t \right)$ and $V\left( t \right)$ are the densities of target cells and virus particles, respectively, at time $t$. The parameter $\beta$ and $\delta$ are the rate constant for infection of target cells by virus and the death rate of virus producing cells, respectively. The combined parameter $r = p\beta /c$ represents the viral replication rate per target cell, where $p$ and $c$ are the virus production rate of a virus producing cell and the clearance rate of virus particles, respectively. Thanks to this simplification the model has only 5 parameters. The novel model reasonably captures de novo infection process, and these 5 parameters can be estimated more reliably than the parameters of the previous models [14–16].

To quantitatively assess the effect of Vpu on viral spread in vivo, here we used the simplified model of Eqs. (1, 2), and applied this to time course data of the number of CD4^+^ T cells per ml of peripheral blood (PB) and the viral RNA load per ml of plasma of infected humanized mice [8]. We infected 9 and 10 humanized mice with CCR5-tropic wild-type (WT) HIV-1 (strain AD8) [17] and *vpu*-deficient HIV-1 (HIV-1Δ*vpu*) [6, 18], respectively, and 100 μl of PB was routinely collected at 0, 3, 7, 14, and 21 days postinfection, as previously described [8, 19–21]. The amount of viral RNA in 50 μl of plasma was quantified by real-time RT-PCR (Bio Medical Laboratories, Inc). Since memory CD4^+^ T cells are the major population of target cells, their densities were measured by hematometry and flow cytometry as previously described [8, 19–21]. Briefly, the number of human leukocytes in 10 μl of PB was measured by using a Celltac α MEK-6450 (Nihon Kohden, Co.), and the percentage of memory CD4^+^ T cells (i.e., CD45^+^ CD3^+^ CD4^+^ CD45RA^−^ cells; target cells) in human CD45^+^ leukocytes was analyzed by flow cytometry using a FACSCanto II (BD Biosciences).

**Fig. 1:**
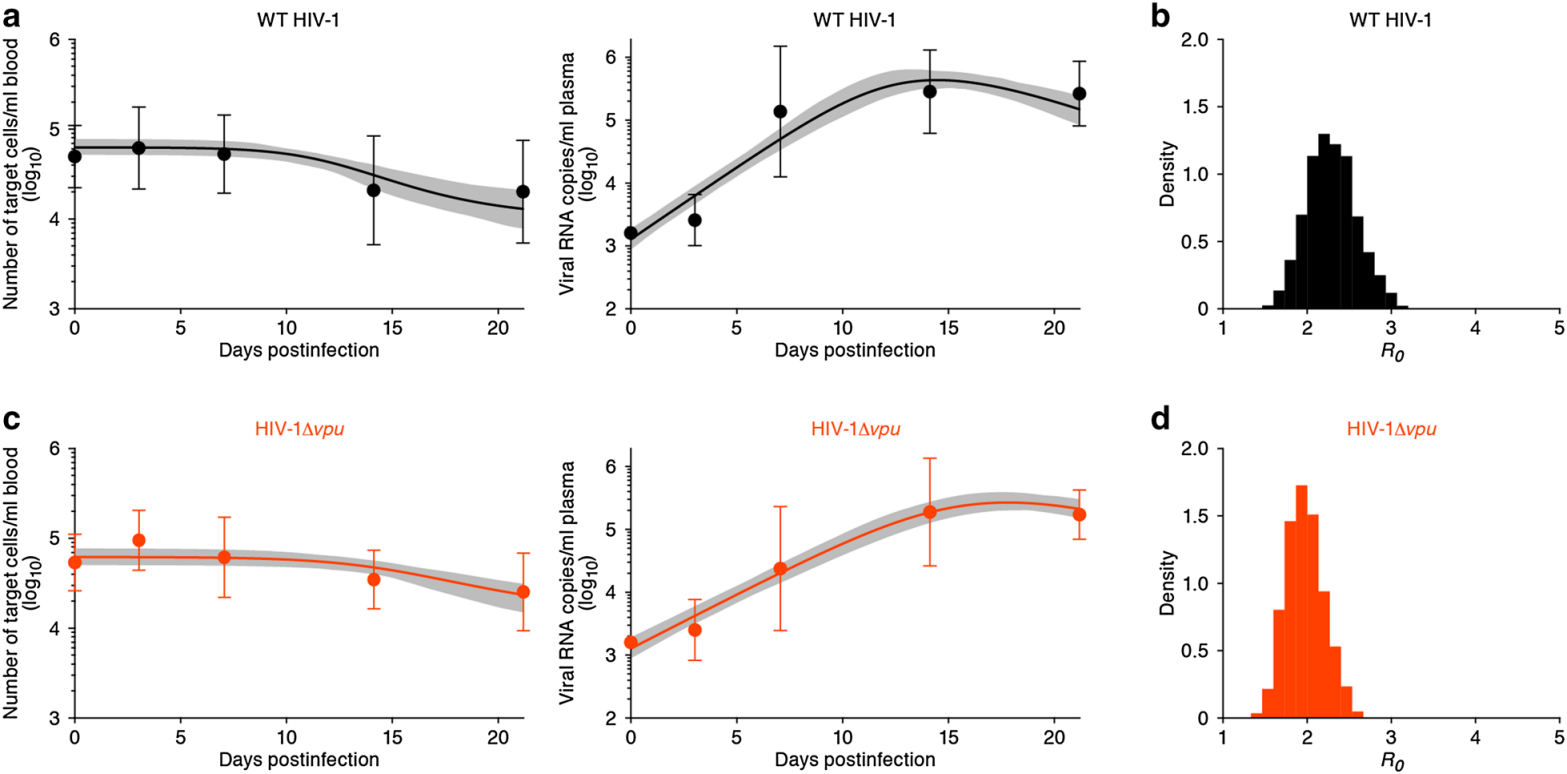
Variability of virus dynamics and basic reproduction number in HIV-1 and HIV-1Δ*vpu* infected humanized mice. The predicted variability of the dynamics of target cells (*left*) and viral loads (*right*) of WT HIV-1 (**a**) and HIV-1Δ*vpu* (**c**) are shown based on Bayesian estimation for the whole datasets using MCMC sampling. The *gray regions* correspond to 95 % posterior predictive intervals. The *solid lines* give the best-fit solution for Eqs. (1, 2), and the *bullets with error bars* show the average with standard deviations. Note that the initial viral loads are set at the detection limit for all samples. The distributions of calculated $R_{0}$ from all accepted MCMC parameter estimates for WT HIV-1 and HIV-1Δ*vpu* are shown in **b** and **d**, respectively. For each *plot*, the last 7000 MCMC samples among the total 10,000 samples are used

Hereafter, we used the whole datasets from 9 WT (i.e., *vpu*-proficient) HIV-1-infected mice and 10 HIV-1Δ*vpu*-infected mice. To assess the variability of kinetic parameters (see Additional file 1), we performed Bayesian estimation for the whole dataset using Markov Chain Monte Carlo (MCMC) sampling. To reduce the number of parameters, we allowed only the parameter *r* to vary between the two groups, and let all other parameters be shared between WT HIV-1 and HIV-1Δ*vpu*-infected mice. In addition, we allowed for broad variations in terms of the measurement error of viral load among the mouse samples into the parameter estimation via MCMC computation (i.e., the variance of the error distribution to be minimized is not constant as is typically assumed in the nonlinear least square method, c.f., [22]). The dynamics of target cells (i.e., memory CD4^+^ T cells) and viral load of WT HIV-1 and HIV-1Δ*vpu* produced with the best fit parameter values are shown in Fig. 1a, c, respectively. These results revealed that the Bayesian inference works well because the model describes the acute phase of WT HIV-1 and HIV-1Δ*vpu* infections in humanized mice reasonably well (c.f. [12, 13]). The gray regions correspond to 95 % posterior predictive intervals, the solid lines give the best-fit solution (mean) for Eqs. (1, 2), and the black and orange dots with bars show the average data with the standard deviations. We summarized the kinetic parameters estimated by the Bayesian inference in Table 1. The marginal posterior distributions for each estimated parameter are shown in Additional file 2, together with scatter plots of paired parameters. Although the ranges of these posterior distributions were relatively narrows, they were not identifiable because *r*, $\delta$, and $T\left( 0 \right)$ correlate with one another. We also fitted our model to the individual data from each of the 9 and 10 humanized mice infected with WT HIV-1 and HIV-1Δ*vpu*, respectively [using the FindMinimum package of *Mathematica 9.0* to minimize the sum of squared residuals (see Additional files 2, 3, 5)]. Not surprisingly, this revealed that the two methods gave very consistent estimates for the parameters underlying WT HIV-1 and HIV-1 Δ*vpu* infection in humanized mice.

**Table 1:** Parameters values and derived quantities for the humanized mouse experiments by the Bayesian inference

Strain [best fit value (95 % CI)]	$T\left( 0 \right)$ (cells/ml)	$V\left( 0 \right)$ (RNA copies/ml)	$\beta$ [(virion/ml)^−1^ day^−1^ (×10^−7^)]	$\delta$ (day^−1^)	$r$ ^†^ [(cell/ml)^−1^ day^−1^ (×10^−6^)]	$R_{0}$ ^‡^ (–)
WT HIV-1	84,621 (66,301–105,897)	1324 (925–1837)	4.41 (2.58–6.78)	0.428 (0.261–0.699)	11.50 (7.79–16.84)	2.29 (1.75–2.90)
HIV-1Δ*vpu*					9.97 (6.59–15.06)	1.97 (1.58–2.44)

^†^ $p = 0.138$ by the repeated bootstrap *t* test and Cohen’s $d = 0.664$ (statistical power $= 0.290$) between WT HIV-1 and HIV-1Δ*vpu*. 10 parameter sets are sampled from the posterior predictive distribution

^‡^ $p = 0.035$ by the repeated bootstrap *t* test and Cohen’s $d = 1.395$ (statistical power $= 0.839$) between WT HIV-1 and HIV-1Δ*vpu*. 10 parameter sets are sampled from the posterior predictive distribution

To reduce the effects of the limited identifiability of $r$, $\delta$, and $T\left( 0 \right)$, we combined them into a single parameter $R_{0}$. The basic reproductive number $R_{0} = rT\left( 0 \right)/\delta$ is a well known quantity which is defined as the average number of newly infected cells produced from any one infected cell, under conditions where most of the target cells are uninfected [12, 13]. For the mice which had enough data to estimate the death rate of infected cells, we directly calculated $R_{0}$ using individual estimation of $T\left( 0 \right),  r$ and $\delta$. Additionally, we calculated $R_{0}$ using the previously estimated mean death rate of $\delta = 0.6$ per day (see Additional files 3, 4, 5). The average basic reproductive number of WT HIV-1 and HIV-1Δ*vpu* in humanized mice is $R_{0} = 2.09 \pm 0.78$ (${\text{mean}} \pm {\text{standard deviation}}$) and $1.72 \pm 0.35$, respectively. Interestingly, although *vpu* is not an essential gene for HIV-1 replication [8], we found that the average of the estimated $R_{0}$ of WT HIV-1 tends to be somewhat greater than that of HIV-1Δ*vpu*. We used all accepted MCMC parameter estimates from the whole datasets, and calculated that the mean values and the 95 % CIs of $R_{0}$ for WT HIV-1 and HIV-1Δ*vpu* are $2.43$ (95 % CI 1.78–3.26) and 2.25 (95 % CI 1.36–3.76), respectively (see Table 1). The distributions of calculated $R_{0}$ for WT HIV-1 and HIV-1 Δ*vpu* are shown in Fig. 1b, d, respectively. Despite the small difference, the mean value of $R_{0}$ for WT HIV-1 is significantly larger than that of that of HIV-1Δ*vpu* ($P < 0.01$ by the repeated bootstrap *t* test).

The retention of virion anchored by tetherin on the surface of infected cells should be reflected by the virus production rate, *p*, in the basic model (see [10, 11]). In addition to its effects on tetherin, Vpu degrades CD4 molecules on the surface of infected cells, and this function of Vpu is highly conserved in pandemic HIV-1 [4, 23–25]. The degradation of the viral receptor CD4 likely prevents aberrant interactions between newly synthesized CD4 molecules and novel viral envelope glycoproteins in the endoplasmic reticulum of infected cells [26–29]. Hence Vpu is expected to increase the number of infectious particles produced per infected cell. We have recently shown that Vpu is closely associated with the down-modulation of CD4 molecules from the infected cells during the acute phase of infection in vivo [8]. Therefore, one could argue that the infection rate $\beta$ in the basic model also decreases in the absence of Vpu. It remains unclear whether this additional effect of Vpu plays a major quantitative role, but fortunately both effects are combined in the composed parameter $r = p\beta /c$ of our model. Hence, the difference in the estimated parameters, especially for the basic reproduction number ($R_{0} = rT\left( 0 \right)/\delta$), between WT HIV-1 and HIV-1Δ*vpu* infection reflects the overall role of Vpu in humanized mouse model.

In a previous study we compared the viral loads of WT HIV-1 with HIV-1Δ*vpu* at different time points in bone marrow (BM) and spleen [8], and observed differences in the viral load only at the earliest time point in the spleen. The amount of cell-free virions in spleen is 100–10,000 fold higher than that in BM [8]. These results suggest that the main source of viral particles in infected humanized mice is the spleen. This is in good agreement with the fact that we now establish that these two viruses differ in the replication rate, allowing a WT virus to approach the set-point viral load in the spleen at an earlier point in time. It could be that the set-point viral load is not strongly dependent on the replication rate (as set-points are also determined by innate immune responses and target cell availability), or that during chronic infection the virus replicates by cell-to-cell infection, which depends less on tetherin [8]. In addition, it is well known that the main target of HIV-1 infection is human CD4^+^ T cells and monocytes/macrophages. In this regard, we have previously reported that human CD4^+^ T cells are relatively frequent in spleen, while monocytes/macrophages are more frequent in BM [19]. Since the current data were collected from the blood, we still do not understand why the approach to set-point is faster in the BM than in the spleen, in terms of kinetics. The evaluation of tissue-specific effects of Vpu/tetherin on viral pathogenesis/replication requires further investigation, e.g., using a spatial model of viral replication [30].

To the best of our knowledge, this is the first report quantifying the role of Vpu in viral spread in vivo. Our findings suggest that the ability of Vpu to down-regulate both tetherin and CD4 moderately increases HIV-1 replication during acute infection. Since the initial viral replicative capacity plays an important role in the subsequent course of disease [31], this moderate increase could still have a large effect on the progression to AIDS.

## Additional files


10.1186/s12977-016-0252-2 Supplementary Materials and Methods. Fitting model to time course experimental data.

10.1186/s12977-016-0252-2 Supplementary Figures. Posterior distributions for each estimated parameter with pairwise scatter plots.

10.1186/s12977-016-0252-2 Supplementary Results. Parameter estimation for individual data from infected humanized mouse with WT HIV-1 and HIV-1Δ*vpu*.

10.1186/s12977-016-0252-2 Figures for Supplementary Results. Dynamics of WT HIV-1 and HIV-1Δ*vpu* infections in humanized mice.

10.1186/s12977-016-0252-2 Tables for Supplementary Results. Parameters values, initial values and derived quantities for the humanized mice infected with the WT HIV-1 and HIV-1Δ*vpu*.

## Abbreviations

HIV-1: human immunodeficiency virus type-1
Vpu: viral protein U
HIV-1 Δ*vpu*: *vpu*-deficient HIV-1
PB: peripheral blood
MCMC: Markov Chain Monte Carlo
