# Supplementary material for: Quantifying the effect of Vpu on the promotion of HIV-1 replication in the humanized mouse model
Source: Retrovirology. 2016 Apr 18;13:23. doi: 10.1186/s12977-016-0252-2 (PMC4834825; doi:10.1186/s12977-016-0252-2)
Supplement: Supplementary file 1 — 10.1186/s12977-016-0252-2 Supplementary Materials and Methods. Fitting model to time course experimental data. [file 12977_2016_252_MOESM1_ESM.docx]

**Additional file 1: Supplementary Materials and Methods**

- 1. Derivation of the novel model from the basic model

After primary infection with HIV and simian immunodeficiency virus, the number of virus particles in the circulation increases rapidly, and after reaching a peak, the viral load declines until it approaches the so-called set point level. The acute phase of virus infection is typically described by using the following “basic model” of virus infection,

$$T'\left( t \right)=-\beta T\left( t \right)V\left( t \right), (S1)$$

$$I'(t)=\beta T\left( t \right)V\left( t \right)-\delta I\left( t \right), (S2)$$

$$V^{'}\left( t \right)=pI\left( t \right)-cV\left( t \right), (S3)$$

where $T(t), I(t),$ and $V(t)$ are the densities of target cells, virus producing cells, and virus particles, respectively, at time $t$. The parameters $\beta, \delta, p$ and $c$ represent the rate constant for infection of target cells by virus, the death rate of virus producing cells, the virus production rate of a virus producing cell, and the clearance rate of virus particles, respectively. Note that, since during acute infection the normal production and loss of target cells is much smaller than the loss due to viral infection, we ignore the effects of normal turnover on the target cell number [[1-3](#_ENREF_1)].

This model can be simplified further by a quasi-steady state (QSS) approximation for the viral particles. Since, the clearance rate of viral particles, $c$, is typically much larger than the death rate, $\delta$, of the infected cells, we can make a QSS assumption, $V^{'}\left( t \right)=0$, and replace Eq. (S3) by $V(t)=pI(t)/c$. Because we fit viral loads, $V(t)$, rather than the number of infected cells, $I(t)$, we also substitute $I\left( t \right)=cV(t)/p$ into Eq. $\left( S2 \right)$ to obtain

$$V'(t)=rT\left( t \right)V\left( t \right)-\delta V\left( t \right), (S4)$$

where $r=p\beta/c$ is the viral replication rate per target cell, and $\delta$ is the death rate of infected cells. Eqs. $\left( S1 \right)$ and $\left( S4 \right)$ together form our novel model, that is, Eqs. (1)(2) in our main text. This model has 5 parameters: $\beta, r, \delta, T\left( 0 \right),$ and $V\left( 0 \right)$, and these were estimated by fitting the CD4^+^ memory T cell data, and the viral load data simultaneously to the model (see below).

- 1. Nonlinear least square method for parameter estimation

For the parameter estimation on individual data from infected humanized mouse, a nonlinear least-square fit was performed simultaneously against the CD4^+^ memory T cell data, and the viral load data using the *Mathematica* function FindMinimum to minimize the following objective function:

$$SSR=\sum_{i=1}^{5} \left\{ \log T\left( t_{i} \right)-\log T_{j}^{e}\left( t_{i} \right) \right\}^{2}+\sum_{i=1}^{5} \left\{ \log V\left( t_{i} \right)-\log V_{j}^{e}\left( t_{i} \right) \right\}^{2}$$

where $T\left( t_{i} \right)$ and $V\left( t_{i} \right)$ are the model-predicted values for target cells and RNA viral load, given by the solution of Eqs.(1)(2) at measurement time $t_{i}$ ($t_{i}=0, 3, 7, 14, 21 d$). The variables with superscript “$e$” are the corresponding experimental measurements of those quantities.

For the parameter estimation on the whole data from infected humanized mice, the same method was applied using the GNU R function [[4](#_ENREF_4)] constrOptim() with the Nelder-Mead method to minimize the following objective function:

$${SSR}^{W}=\sum_{i=1}^{5} \sum_{j=1}^{\#ID} \left\{ \log T\left( t_{i} \right)-\log T_{j}^{e}\left( t_{i} \right) \right\}^{2}+\sum_{i=1}^{5} \sum_{j=1}^{\#ID} \left\{ \log V\left( t_{i} \right)-\log V_{j}^{e}\left( t_{i} \right) \right\}^{2},$$

where $T\left( t_{i} \right)$ and $V\left( t_{i} \right)$ are the model-predicted values for target cells and RNA viral load from ID-*j* mouse, given by the solution of Eqs.(1)(2) at measurement time $t_{i}$ ($t_{i}=0, 3, 7, 14, 21 d$). The variables with superscript “$e$” are the corresponding experimental measurements of those quantities for ID-*j* humanized mouse. 95% CI for the estimated parameters was constructed by the non-parametric bootstrap method [[5](#_ENREF_5)]. More precisely, sampling of time points with replacement was performed in each bootstrap replicate from the total datasets which are indexed by the combination of measurement time $t_{i}$ ($t_{i}=0, 3, 7, 14, 21 d$) and mouse ID $j\in\left\{ 1, 2,\cdots, 9 \right\}$ or $j\in\left\{ 10, 11,\cdots, 19 \right\}$ (cf. similar procedure was adopted in [[6](#_ENREF_6)]). To account for potential bias in bootstrap estimates and non-normality of bootstrapped parameter distributions, the bias-corrected accelerated (BCa) confidence interval was adopted to obtain the both-sided 95% CI from 10,000 times bootstrap replicates (see **Table S1** and **S2** in **Additional file 5**).

- 1. Bayesian interference for parameter estimation

In the MCMC computation, the R package FME [[7](#_ENREF_7)] was used to generate Markov chains. The FME package adopts essentially the same assumptions as conventional parameter estimations, namely, a measurement error between model prediction and experimental data obeys a Gaussian distribution with mean 0 and a constant variance. The FME package enables us to incorporate broad variations in terms of the measurement error of viral load among the mouse samples into parameter estimation. More precisely, in the FME package, it is postulated that the inverse of variance of the error distribution is not constant but Gamma distributed. Moreover, for prior of parameter distributions, a Gaussian is postulated (see [[7](#_ENREF_7)] for more detailed explanation). The “delayed rejection and adaptive Metropolis algorithm” [[8](#_ENREF_8)] was adopted in our MCMC computation that is provided as the default scheme in the FME package to ensure the convergence of Malkov chains.

We estimated the posterior distribution via MCMC computation using the function modMCMC() in the FME package. In our computations, 10,000 Malkov chains are generated. The first 3000 chains are discarded as burn-in samples. The mean value was adopted as a representative value of each estimated parameter distribution. The 2.5% and 97.5% percentiles were calculated as the 95% CI (credible interval) as shown in **Table 1**. Estimated posterior distributions with pairwise scatter plots were drawn in **Figure**s **A** and **B** in **Additional file 2**. To obtain the distribution of basic reproduction number $R_{0}=rT\left( 0 \right)/\delta$, 7000 accepted Malkov chains were used for calculation. Since a large number of parameters are sampled (i.e., 7000), small numerical values of $\delta$ can be chosen despite of their small probabilities to be sampled. Then extraordinary values of $R_{0}=rT\left( 0 \right)/\delta$ in which $\delta$ is in the denominator are artificially generated. To avoid bias in the estimation of the mean value, the estimated distribution of $R_{0}$ was trimmed by eliminating values which fall more than 1.5 times the interquartile range as outlier. To take the possibilities of non-normality and heteroscedasticity into account, the bootstrap *t*-test [[5](#_ENREF_5)] was performed to determine whether the means of $R_{0}$ for WT HIV-1 and HIV-1 Δ*vpu* are statistically significantly different. In each bootstrap *t*-test, 100 among 7000 values are sampled to calculate the *t*-statistics for WT HIV-1 and HIV-1Δ*vpu*. In one procedure, 10,000 bootstrap replicates were generated to calculate the *p* value. This procedure was repeated 100 times to obtain an average *p* value. The average *p* value was calculated as 0.008898. All results can be reproduced by using the same random seed.

**REFERENCES**

1. Davenport MP, Zhang L, Shiver JW, Casmiro DR, Ribeiro RM, Perelson AS: **Influence of peak viral load on the extent of CD4+ T-cell depletion in simian HIV infection.** *J Acquir Immune Defic Syndr* 2006, **41:**259-265.

2. Wilson DP, Mattapallil JJ, Lay MD, Zhang L, Roederer M, Davenport MP: **Estimating the infectivity of CCR5-tropic simian immunodeficiency virus SIV(mac251) in the gut.** *J Virol* 2007, **81:**8025-8029.

3. Petravic J, Ribeiro RM, Casimiro DR, Mattapallil JJ, Roederer M, Shiver JW, Davenport MP: **Estimating the impact of vaccination on acute simian-human immunodeficiency virus/simian immunodeficiency virus infections.** *J Virol* 2008, **82:**11589-11598.

4. Team RC: **R: a language and environment for statistical computing. Vienna, Austria: R Foundation for Statistical Computing; 2012.** *Open access available at:* [*http://cran*](http://cran) *r-project org* 2014.

5. Efron B, Tibshirani RJ: *An introduction to the bootstrap.* CRC press; 1994.

6. Ganusov VV, Milutinovic D, De Boer RJ: **IL-2 regulates expansion of CD4+ T cell populations by affecting cell death: insights from modeling CFSE data.** *J Immunol* 2007, **179:**950-957.

7. Soetaert K, Petzoldt T: **Inverse modelling, sensitivity and monte carlo analysis in R using package FME.** *Journal of Statistical Software* 2010, **33**.

8. Haario H, Laine M, Mira A, Saksman E: **DRAM: efficient adaptive MCMC.** *Statistics and Computing* 2006, **16:**339-354.
