# Supplementary material for: Quantifying the effect of Vpu on the promotion of HIV-1 replication in the humanized mouse model
Source: Retrovirology. 2016 Apr 18;13:23. doi: 10.1186/s12977-016-0252-2 (PMC4834825; doi:10.1186/s12977-016-0252-2)
Supplement: Supplementary file 2 — 10.1186/s12977-016-0252-2 Supplementary Figures. Posterior distributions for each estimated parameter with pairwise scatter plots. [file 12977_2016_252_MOESM2_ESM.docx]

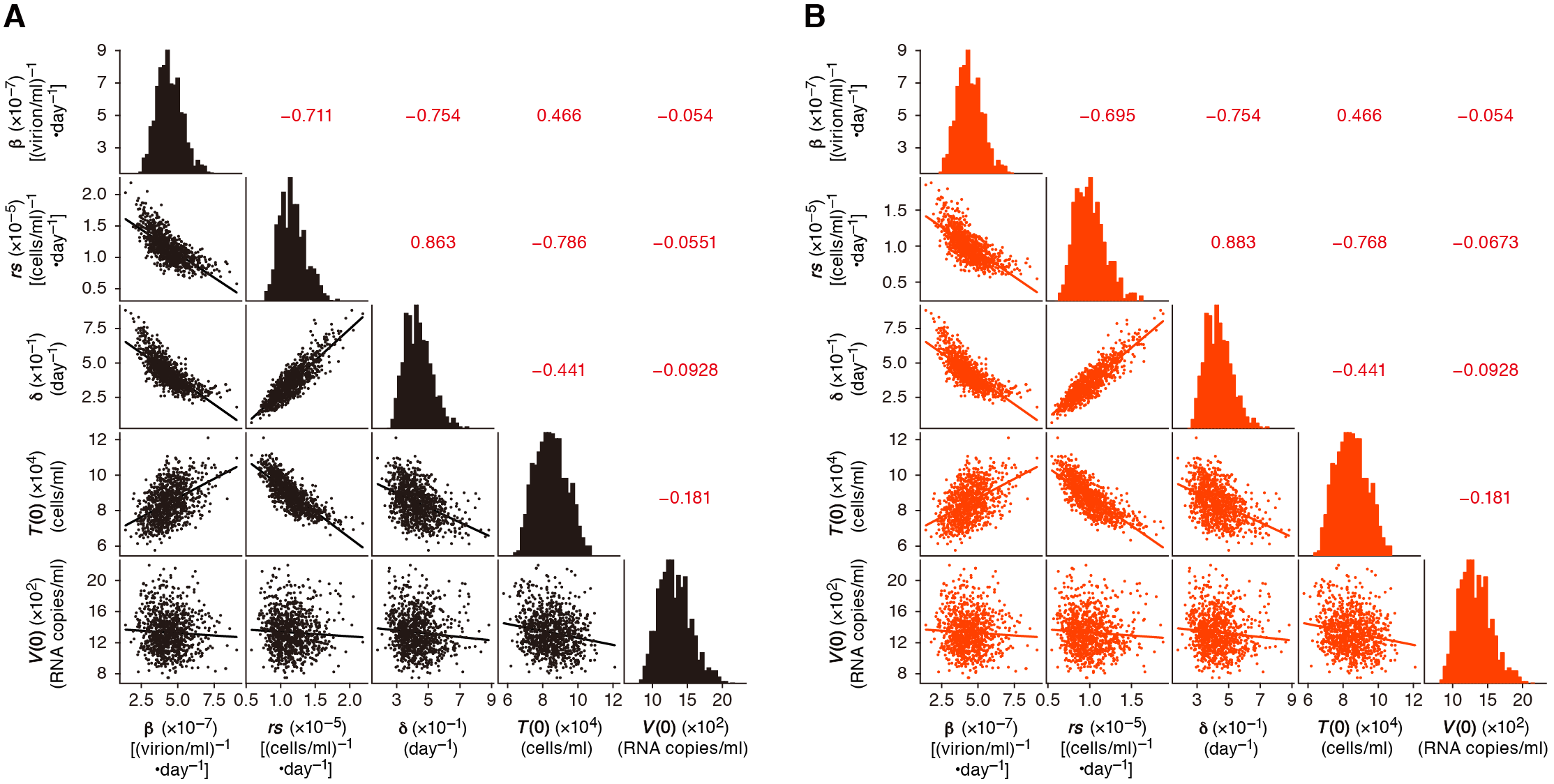
**Additional file 2: Supplementary Figures**

**Figure S1. Posterior distributions for the estimated parameters with pairwise scatter plots.** Each distribution on the diagonal represents the posterior distribution of a parameter, based upon the last 7,000 MCMC samples of the total of 10,000 samples. Each panel in the lower-diagonal block represents a pairwise scatter plot with a linear regression line. The correlation coefficients between the pairwise parameter distributions are shown in the upper-diagonal block. Panels (A) and (B) represent computed results for WT HIV-1 and HIV-1Δ*vpu* infection, respectively.
