# Supplementary material for: Quantifying the effect of Vpu on the promotion of HIV-1 replication in the humanized mouse model
Source: Retrovirology. 2016 Apr 18;13:23. doi: 10.1186/s12977-016-0252-2 (PMC4834825; doi:10.1186/s12977-016-0252-2)
Supplement: Supplementary file 3 — 10.1186/s12977-016-0252-2 Supplementary Results. Parameter estimation for individual data from infected humanized mouse with WT HIV-1 and HIV-1Δvpu. [file 12977_2016_252_MOESM3_ESM.docx]

**Additional file 3: Supplementary Results**

We employed the solution of Eqs.(1)(2) to fit the 21 day time courses of viral loads and target cells as observed in the 9 and 10 humanized mice infected with WT HIV-1 and HIV-1Δ*vpu*, respectively. The estimated parameter and initial values of each infected individual mouse with WT HIV-1 and HIV-1Δ*vpu* are given in **Table S1** and **S2** in **Additional files 5**, respectively. Usually, the descending slope of the log viral load roughly corresponds to the death rate, $\delta$, of infected cells [[1-4](#_ENREF_1)]. However, for some of the mice, there was insufficient viral load data after the peak, and we could not obtain a realistic estimate of the death rate. For those mice we fixed the death rate $\delta=0.6$ per day, which is the mean value of the previously estimated death rate in [[1](#_ENREF_1)]. Note that, we obtained similar kinetic parameter estimates (see **Table S3** and **S4** in **Additional files 5**), even if we fixed $\delta=0.6$ for all the mice. Averaging the individual estimates, we obtained the infection rate of $\beta=(mean\pm standard deviation, 3.33\pm4.02)\times{10}^{-7}$ and $(4.68\pm5.24)\times{10}^{-7}$ per virion per day, the average viral replication rate of $r=(22.2\pm19.8)\times{10}^{-6}$ and $(11.5\pm6.43)\times{10}^{-6}$ per target cell per day, and the death rate of $\delta=0.60\pm0.13$ and $0.66\pm0.14$ per day, for the WT HIV-1 and HIV-1Δ*vpu*, respectively. The behavior of the model using the best-fit parameter estimates is shown together with the individual data in **Figure**s **A** and **C** in **Additional files 4**, which reveals that the model describes the acute phase of WT HIV-1 and HIV-1Δ*vpu* infections in humanized mice reasonably well (see also [[1](#_ENREF_1), [5](#_ENREF_5)]). We also use the whole datasets from the 9 and 10 infected mice with WT HIV-1 and HIV-1Δ*vpu* to fit our model. We obtained very similar estimates of $\beta=3.32\times{10}^{-7}$ (95% bootstrap bias-corrected accelerated (BCa) confidence interval (95% CI) : $1.10-7.78\times{10}^{-7}$) and $5.30\times{10}^{-7}$ (95% CI : $2.13-11.78\times{10}^{-7}$) per virion per day, $r=13.22\times{10}^{-6}$ (95% CI: $8.70-28.40\times{10}^{-6}$) and $9.31\times{10}^{-6}$ (95% CI : $5.08-18.54\times{10}^{-6}$) per target cell per day, and $\delta=0.48$ (95% CI : $0.29-1.21$) and $0.42$ (95% CI : $0.14-1.01$) per day for WT HIV-1 and HIV-1Δ*vpu*, respectively, comparing with the individual estimates (see **Table S1** and **S2** in **Additional files 5**). The behavior of the model using the best-fit parameter estimates is shown together with the whole data of WT HIV-1 and HIV-1Δ*vpu* infection in **Figure**s **B** and **D** in **Additional files 4**, respectively.

**REFERENCES**

1. Ikeda H, de Boer RJ, Sato K, Morita S, Misawa N, Koyanagi Y, Aihara K, Iwami S: **Improving the estimation of the death rate of infected cells from time course data during the acute phase of virus infections: application to acute HIV-1 infection in a humanized mouse model.** *Theor Biol Med Model* 2014, **11:**22.

2. Little SJ, McLean AR, Spina CA, Richman DD, Havlir DV: **Viral dynamics of acute HIV-1 infection.** *J Exp Med* 1999, **190:**841-850.

3. Nowak MA, Lloyd AL, Vasquez GM, Wiltrout TA, Wahl LM, Bischofberger N, Williams J, Kinter A, Fauci AS, Hirsch VM, Lifson JD: **Viral dynamics of primary viremia and antiretroviral therapy in simian immunodeficiency virus infection.** *J Virol* 1997, **71:**7518-7525.

4. Ribeiro RM, Qin L, Chavez LL, Li D, Self SG, Perelson AS: **Estimation of the initial viral growth rate and basic reproductive number during acute HIV-1 infection.** *J Virol* 2010, **84:**6096-6102.

5. Ikeda H, Nakaoka S, Sato K, Misawa N, Koyanagi Y, Iwami S: **Effect of eclipse phase on quantifying viral dynamics of acute HIV-1 infection in humanized mouse model.** *Nonlinear Theory and Its Applications, IEICE* 2015, **6:**47-53.
