# Supplementary material for: Quantifying the effect of Vpu on the promotion of HIV-1 replication in the humanized mouse model
Source: Retrovirology. 2016 Apr 18;13:23. doi: 10.1186/s12977-016-0252-2 (PMC4834825; doi:10.1186/s12977-016-0252-2)
Supplement: Supplementary file 4 — 10.1186/s12977-016-0252-2 Figures for Supplementary Results. Dynamics of WT HIV-1 and HIV-1Δvpu infections in humanized mice. [file 12977_2016_252_MOESM4_ESM.docx]

**Additional file 4: Figures for Supplementary Results**


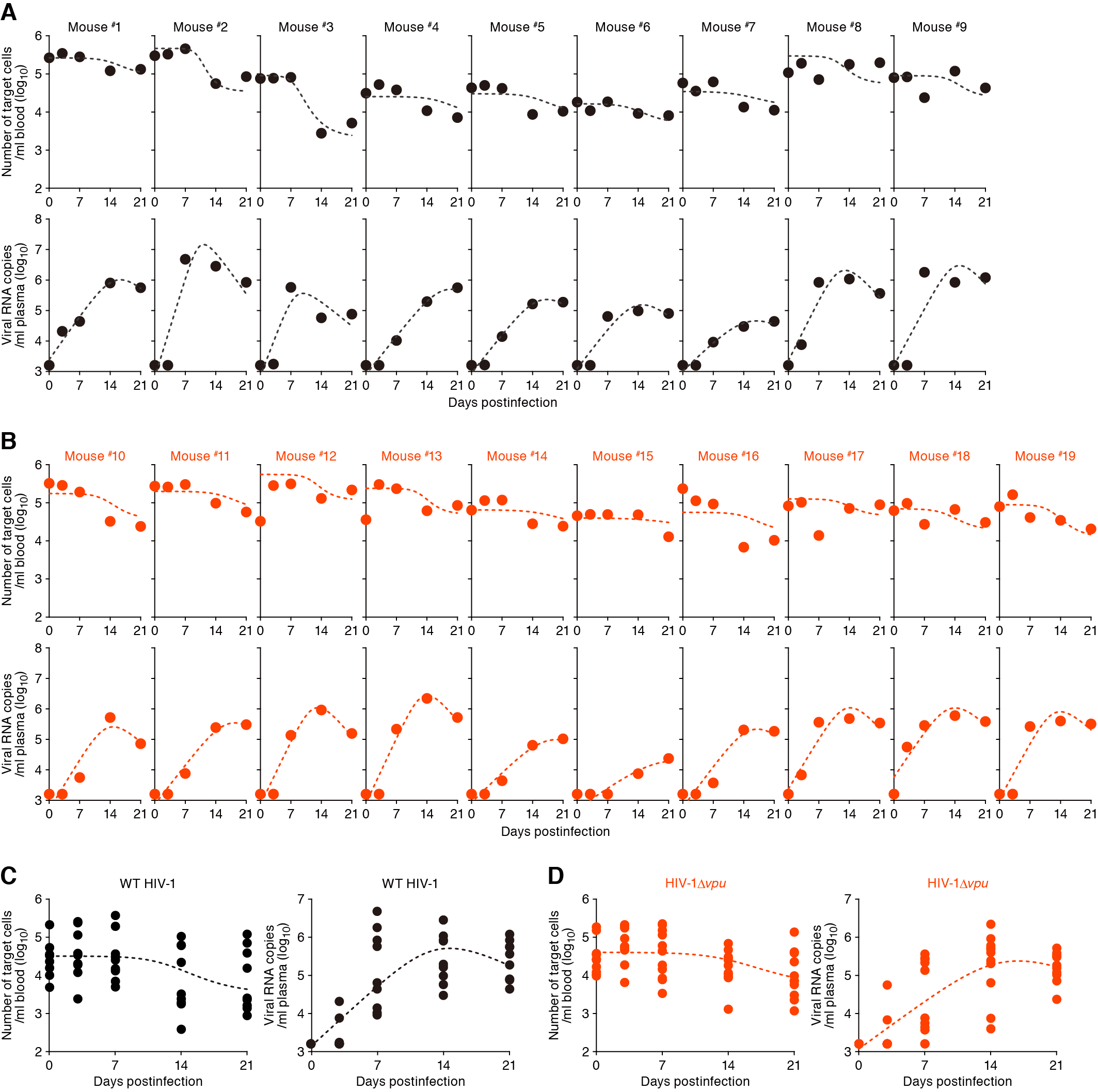


**Figure S2. Dynamics of WT HIV-1 and HIV-1Δ*vpu* infections in humanized mice.** The number of CD4^+^ T cells in per ml of PB (top) and the viral RNA load per ml of plasma (bottom) for 9 humanized mice infected with WT HIV-1 **(A)** and 10 mice infected with HIV-1Δ*vpu* **(C)**. The average number of CD4^+^ T cells (left) and the average viral RNA load (right) of 9 humanized mice infected with WT HIV-1 **(B)** and 11 mice infected with HIV-1Δ*vpu* **(D)**. The black and orange symbols denote these time course data and the solid lines depict the best fit of Eqs.(1)(2) to the data from each individual.
