# Supplementary material for: Quantifying the effect of Vpu on the promotion of HIV-1 replication in the humanized mouse model
Source: Retrovirology. 2016 Apr 18;13:23. doi: 10.1186/s12977-016-0252-2 (PMC4834825; doi:10.1186/s12977-016-0252-2)
Supplement: Supplementary file 5 — 10.1186/s12977-016-0252-2 Tables for Supplementary Results. Parameters values, initial values and derived quantities for the humanized mice infected with the WT HIV-1 and HIV-1Δvpu. [file 12977_2016_252_MOESM5_ESM.docx]

**Additional file 5: Tables for Supplementary Results**

**Table S1. Parameters values and derived quantities for WT HIV-1-infected humanized mice**

| Mouse # | $T(0)$ | $V(0)$ | $\beta$ | $r$ | $\delta$ | $R_{0}$ |
| --- | --- | --- | --- | --- | --- | --- |
|  | cells/ml | RNA copies/ml | (virion/ml)^-1^・day^-1^(×10^-7^) | (cell/ml)^-1^・day^-1^(×10^-6^) | day^-1^ | --- |
| 1 | 274273 | 2461 | 1.02 | 4.45 | 0.75 | 1.62 |
| 2 | 489113 | 652 | 0.32 | 3.37 | 0.58 | 2.83 |
| 3 | 93239 | 717 | 12.4 | 12.4 | 0.30 | 3.88 |
| 4^*^ | 25802 | 798 | 2.00 | 39.3 | 0.60 | 1.69 |
| 5^*^ | 30614 | 884 | 4.37 | 33.2 | 0.60 | 1.69 |
| 6^*^ | 16617 | 1245 | 6.91 | 61.9 | 0.60 | 1.71 |
| 7^*^ | 35126 | 1030 | 1.37 | 26.0 | 0.60 | 1.52 |
| 8 | 307993 | 2248 | 1.02 | 4.12 | 0.61 | 2.07 |
| 9 | 92346 | 1578 | 0.54 | 15.0 | 0.78 | 1.79 |
| Mean^†^ | 151680 | 1290 | 3.33 | 22.2 | 0.60 | 2.09 |
| S.D. ^†^ | 166804 | 669.3 | 4.02 | 19.8 | 0.13 | 0.78 |
| All^‡^ : best fit value | 75880 | 1370 | 3.32 | 13.22 | 0.48 | 2.09 |
| 95% CI | 51850-107103 | 985-2072 | 1.10-7.78 | 8.70-28.40 | 0.29-1.21 | 1.20-2.73 |

^*^The death rate is fixed by $\delta=0.60$ per day.

^†^Averaged value and standard deviation of the individual estimates of mouse #1, …, #9.

^‡^ We used the whole data of the CD4^+^ T cell and viral load from the 9 infected mice with WT HIV-1.

**Table S2. Parameters values and derived quantities for HIV-1Δ*vpu*-infected humanized mice**

| Mouse # | $T(0)$ | $V(0)$ | $\beta$ | $r$ | $\delta$ | $R_{0}$ |
| --- | --- | --- | --- | --- | --- | --- |
|  | cells/ml | RNA copies/ml | (virion/ml)^-1^・day^-1^(×10^-7^) | (cell/ml)^-1^・day^-1^(×10^-6^) | day^-1^ | --- |
| 10 | 180579 | 490 | 6.45 | 6.06 | 0.55 | 1.99 |
| 11^*^ | 206318 | 733 | 3.00 | 4.97 | 0.60 | 1.71 |
| 12 | 402151 | 1431 | 1.86 | 3.10 | 0.67 | 1.86 |
| 13 | 248509 | 805 | 0.78 | 5.67 | 0.71 | 1.99 |
| 14^*^ | 66550 | 847 | 6.68 | 13.9 | 0.60 | 1.54 |
| 15^*^ | 40664 | 525 | 18.2 | 20.3 | 0.60 | 1.38 |
| 16^*^ | 57479 | 590 | 5.12 | 18.1 | 0.60 | 1.73 |
| 17 | 130096 | 2346 | 1.09 | 11.1 | 0.91 | 1.60 |
| 18 | 70164 | 4813 | 0.88 | 19.8 | 0.87 | 1.10 |
| 19 | 91257 | 1107 | 2.71 | 11.6 | 0.46 | 2.32 |
| Mean^†^ | 149377 | 1369 | 4.68 | 11.5 | 0.66 | 1.72 |
| S.D. ^†^ | 113081 | 1332 | 5.24 | 6.43 | 0.14 | 0.35 |
| All^‡^ : best fit value | 89828 | 1189 | 5.30 | 9.31 | 0.42 | 1.99 |
| 95% CI | 71018-112881 | 834-1659 | 2.14-11.78 | 5.08-18.54 | 0.14-1.00 | 1.35-2.96 |

^*^The death rate is fixed by $\delta=0.60$ per day.

^†^Averaged value and standard deviation of the individual estimates of mouse #10, …, #19.

^‡^ We used the whole data of the CD4^+^ T cell and viral load from the 10 infected mice with HIV-1Δ*vpu*.

**Table S3. Parameters values and derived quantities for WT HIV-1-infected humanized mice with fixed values of** $\delta=0.6$

| Mouse # | $T(0)$ | $V(0)$ | $\beta$ | $r$ | $\delta$ | $R_{0}$ |
| --- | --- | --- | --- | --- | --- | --- |
|  | cells/ml | RNA copies/ml | (virion/ml)^-1^・day^-1^(×10^-7^) | (cell/ml)^-1^・day^-1^(×10^-6^) | day^-1^ | --- |
| 1 | 286219 | 2498 | 1.24 | 3.72 | 0.60 | 1.77 |
| 2 | 480135 | 647 | 0.30 | 3.47 | 0.60 | 2.78 |
| 3 | 55192 | 615 | 4.33 | 27.1 | 0.60 | 2.49 |
| 4 | 25802 | 798 | 2.00 | 39.3 | 0.60 | 1.69 |
| 5 | 30614 | 884 | 4.37 | 33.2 | 0.60 | 1.69 |
| 6 | 16617 | 1245 | 6.91 | 61.9 | 0.60 | 1.71 |
| 7 | 35126 | 1030 | 1.37 | 26.0 | 0.60 | 1.52 |
| 8 | 230670 | 2517 | 1.06 | 5.28 | 0.60 | 2.03 |
| 9 | 91708 | 1713 | 0.67 | 13.1 | 0.60 | 2.01 |
| Mean^†^ | 139120 | 1327 | 2.47 | 23.7 | 0.60 | 1.97 |
| S.D. ^†^ | 160483 | 748.1 | 2.23 | 19.6 | 0.00 | 0.42 |

^†^Averaged value and standard deviation of the individual estimates of mouse #1, …, #9.

**Table S4. Parameters values and derived quantities for HIV-1Δ*vpu*-infected humanized mice with fixed values of** $\delta=0.6$

| Mouse # | $T(0)$ | $V(0)$ | $\beta$ | $r$ | $\delta$ | $R_{0}$ |
| --- | --- | --- | --- | --- | --- | --- |
|  | cells/ml | RNA copies/ml | (virion/ml)^-1^・day^-1^(×10^-7^) | (cell/ml)^-1^・day^-1^(×10^-6^) | day^-1^ | --- |
| 10 | 157930 | 510 | 5.92 | 7.17 | 0.60 | 1.89 |
| 11 | 206318 | 733 | 3.00 | 4.97 | 0.60 | 1.71 |
| 12 | 414601 | 915 | 2.28 | 2.95 | 0.60 | 2.04 |
| 13 | 270112 | 801 | 0.92 | 4.84 | 0.60 | 2.18 |
| 14 | 66550 | 847 | 6.68 | 13.9 | 0.60 | 1.54 |
| 15 | 40664 | 525 | 18.2 | 20.3 | 0.60 | 1.38 |
| 16 | 57479 | 590 | 5.12 | 18.1 | 0.60 | 1.73 |
| 17 | 93706 | 2506 | 1.67 | 11.8 | 0.60 | 1.84 |
| 18 | 76710 | 4898 | 1.27 | 14.5 | 0.60 | 1.85 |
| 19 | 82199 | 1099 | 2.07 | 14.6 | 0.60 | 2.00 |
| Mean^†^ | 146627 | 1342 | 4.71 | 11.3 | 0.60 | 1.82 |
| S.D. ^†^ | 119339 | 1377 | 5.15 | 6.00 | 0.00 | 0.24 |

^†^Averaged value and standard deviation of the individual estimates of mouse #10, …, #19.
